# The Diagnosis of Torsion of the Great Omentum, Illustrated by Two Case-Histories

**Published:** 1920-12

**Authors:** James Swain

**Affiliations:** Emeritus Professor of Surgery in the University of Bristol; Surgeon to the Bristol Royal Infirmary


### THE DIAGNOSIS OF TORSION OF THE GREAT OMENTUM, ILLUSTRATED BY TWO CASE-HISTORIES.

BY

James Swain, C.B., M.S., M.D. Lond., F.R.C.S.,

*Emeritus Professor of Surgery in the University of Bristol; Surgeon to the Bristol Royal Infirmary.*

---

In acute abdominal troubles a positive diagnosis is generally admitted to be difficult, and the list of possible causes of the "acute abdomen" is a long one.

The differentiation of Henoch's purpura and intussusception, acute pancreatitis or enterospasm and organic intestinal obstruction, acute tuberculous peritonitis and appendicitis have only to be mentioned to emphasise the snares that beset the path of the surgeon in his endeavour to form an accurate opinion of the etiology of a particular case.

The difficulty of diagnosis is due to the fact that the early symptoms of acute abdominal trouble are generally the same whatever the cause may be. In every case the first symptoms are produced, not by the distinctive characters of the lesion, but by the effect of the lesion on the peritoneal nerves, and usually made evident by a group of symptoms—pain, vomiting and more or less collapse—known as "peritonism" (Treves).. Hence the similarity of symptoms at the commencement of the illness and hence the confusion.

It is because of the infrequency of torsion of the great omentum and the difficulty of its diagnosis that the cases described below are thought worthy of publication.

Torsion of the omentum is so rare that Prescott Hedley[1] was only able to find references to ninety-three cases;

[1] *Brit. M. J.*, 1911, ii. 1,246.

and of seventy-three cases in which he obtained details from the writings of Corner and Pinches, Lejars, Picquet and others, there were only thirteen in which there was no history of a hernia, which was present in almost all the other sixty cases at the time of operation.

**Case 1.**—Mr. N., æt. 58, was seized with sharp pain in the region of the gall-bladder on April 24th. On the following day there was great tenderness and he was slightly jaundiced. By April 29th the pain had become rather more general and appeared to be associated with flatulence. There was no sickness. The bowels had acted freely after purgation and there was no constipation. The gall-bladder was slightly distended, but the presence of muscle guarding made examination difficult. There was no tenderness in the appendix region. On April 25th the temperature was 102°, came down to normal the following day, and rose to 100° on May 3rd. On this date a large "lump" was felt in the neighbourhood of the gall-bladder. On opening the abdomen a dark red, friable mass with yellowish spots was seen adherent to the parietes, liver and adjacent intestines. On detaching this mass it was found to be a matted portion of omentum near the hepatic flexure. This was tied off, the piece removed being about 6″ by 3″ by 2″. The gall-bladder was distended, but no stones were found. The abdomen was closed and the patient made an uninterrupted recovery. The pathological report on the tissue removed was as follows: "This section shows a very congested omentum. There are no foci of tuberculosis, nor any collection of tumour cells. Sections taken from different parts have been examined for various micro-organisms, but without finding any, either Gram-positive or negative. It is possible only to diagnose it as a condition of active hyperæmia, probably occasioned by some adjacent inflammatory process."

**Case 2.**—Mrs. H., æt. 25, had a feeling of general abdominal discomfort, beginning on July 13th. Later there was severe pain, at first near the umbilicus and afterwards in the right iliac fossa with tenderness and muscle guarding. On July 16th the temperature was 102°, on July 18th it was 101°, and on July 19th (when I first saw her) it was 99°. The bowels had acted without difficulty throughout the illness and there was no vomiting. There was no history of any previous attack of a similar character. A definite tender lump about the size of a large walnut could be felt just above a line joining the right anterior superior iliac spine with the umbilicus. An incision over the swelling showed the mass to consist of matted omentum—dark red in colour with yellowish spots—just below the right end of the transverse colon. The affected portion was tied off and removed. The appendix was quite distinct from the swelling; it was, however, removed for safety's sake, but it showed no definite evidence of appendicitis. Recovery was uninterrupted.

The pathological report on the tissue removed was as follows: "The sections show the presence of wide-spread active hyperæmia. I cannot find the signs of actual suppuration in the shape of polynuclear exudate or bacteria, but there is a slight amount of perivascular mononuclear infiltration. There is not any evidence of malignant change or infiltration."

Whether it is correct to describe these cases as "torsion" of the omentum I am not sure, as although the affected omentum was matted, there was no evidence of actual twisting.

Clinically the symptoms were largely of an inflammatory character. The first case resembled an attack of cholecystitis and the second case resembled appendicitis. Probably some local peritonitis was commencing at the time of operation, but it seems clear from the microscopic appearances that the primary changes—as shown by the presence of hyperæmia with an absence of bacteria and polynuclear leucocytes—were of a simple vascular nature and not those of inflammation. Such changes could easily be produced by a slight degree of torsion.

In the present state of our knowledge it seems probable that such cases are not likely to be definitely diagnosed before operation; but it is worth bearing in mind that in both of my cases the symptoms developed somewhat slowly, vomiting was absent throughout, and there was no interference with the action of the bowels.

Operative procedure is necessary, and in this we have one of the many examples of the fact that the treatment of acute abdominal troubles has advanced beyond our powers of diagnosis.

